# Friend and Foe

**Published:** 1890-05-17

**Authors:** 


### Passing Topics.

FRIEND AND FOE.

Lord Derby's advocacy of the hospitals comes opportunely at the time that the indictment of the Charity Organization Society is being elaborated before the Select Committee of the House of Lords, and it is impossible to avoid regretting that a peer who has long shown so keen an interest in medical charity, and whose knowledge of its ways and work is so far from being superficial, should not have been included in the committee charged with the inquiry now proceeding.

We cannot, however, doubt for a moment the ability of the committee as it stands to resist the conclusions which the Charity Organization Society doubtless has already in view. On the contrary, we have every right to suppose that Lord Sandhurst and his colleagues may be reckoned upon to secure fair play. None the less the practical knowledge of Lord Derby would have been invaluable, and the more because, however profound his appreciation of the value of the hospitals, he is not to be classed among the downright opponents of the society which figures as complainant in the suit, and moreover, as his recent speech shows, is quite as much alive to the defects of the present system as anybody who has only a single-minded desire to do right can wish him to be.

We repeat, there is no objection to inquiry, but the representatives of institutions which are nothing if they be not benevolent may well be excused for their suspicion of the aims of a society which is notoriously devoid of sympathy with them, and unless institutional opinion is woefully wrong, is ambitious to cripple or extinguish all charitable undertakings which are unwilling to adapt their methods to its requirements. The idea of a "Central Council," as set before the Committee by Colonel Montefiore, appears to be a deliberate proposal to bring the great hospitals of London under the control of the society he represents, and with all due respect to rightful authority, it may be said at once that is precisely what the hospitals will not submit to.

It might be pertinently asked of Colonel Montefiore and his fellow thinkers what it is which entitles them to pose as the censors of our hospitals? It cannot be the profundity of their knowledge, else they would remember that they are proposing to deal with institutions in chief part voluntarily maintained, and that to offend the susceptibilities of their supporters will merely have the effect of stopping the supplies.

The best friends of hospitals are desirous of needful reform, and it will be to the interest of the hospitals themselves to assist in the correction of certain evils which have crept in. But it is one thing to demand that hospital work shall be improved and rendered, as far as possible, free from defect, and quite another to attempt to eliminate that philanthropic and sympathetic element which is its mainspring and glory, and the feature especially repugnant to a society whose whole history is a protest against prompt and ungrudging benevolence. If we are to have a régime of charity attenuation in respect of our hospitals, with its superabundance of inquiries, reports, certificates, references, hesitations, and ultimate refusals, Heaven help the sick poor!

The hospitals are just now the targets at which every small economist and social reformer—everybody in fact who has done nothing to help them, and is blind to their splendid record—is ready to aim a shaft. Unfortunately the hospitals have no common organisation, and this it is which helps their shortcomings and weakens their defence. It is a timely reminder of Lord Derby's that "the illness of a patient is a fact" and that "of all charities, hospitals are least likely to be abused." This, too, was the opinion of old Samuel Johnson, and it is as true now as in his day. The remedy of such abuses as there are must be entrusted to friendly hands, and might almost have been left for application to the hospitals themselves, were it not that the very first requirement is the absorption of superfluous and the suppression of pernicious institutions masquerading under the name of hospitals. What the legitimate hospitals have to bear in mind at present is that a hand is raised apparently to smite them rather than to right them, and Lord Sandhurst's Committee will need all the assistance they can give if good and not evil is to ensue.

Agricola.

